# Neighborhood socioeconomic position and tuberculosis transmission: a retrospective cohort study

**DOI:** 10.1186/1471-2334-14-227

**Published:** 2014-04-27

**Authors:** Eyal Oren, Masahiro Narita, Charles Nolan, Jonathan Mayer

**Affiliations:** 1 Division of Epidemiology & Biostatistics, University of Arizona, Tucson, AZ, USA; 2 Department of Epidemiology, University of Washington, Seattle, WA, USA; 3 Public Health-Seattle & King County Tuberculosis Control Program, Seattle, WA, USA; 4 Division of Pulmonary and Critical Care Medicine, University of Washington, Seattle, WA, USA

**Keywords:** Tuberculosis, Genotyping, Socioeconomic status, Infectious disease transmission, Multilevel, Molecular epidemiology

## Abstract

**Background:**

Current understanding of tuberculosis (TB) genotype clustering in the US is based on individual risk factors. This study sought to identify whether area-based socioeconomic status (SES) was associated with genotypic clustering among culture-confirmed TB cases.

**Methods:**

A retrospective cohort analysis was performed on data collected on persons with incident TB in King County, Washington, 2004–2008. Multilevel models were used to identify the relationship between area-level SES at the block group level and clustering utilizing a socioeconomic position index (SEP).

**Results:**

Of 519 patients with a known genotyping result and block group, 212 (41%) of isolates clustered genotypically. Analyses suggested an association between lower area-based SES and increased recent TB transmission, particularly among US-born populations. Models in which community characteristics were measured at the block group level demonstrated that lower area-based SEP was positively associated with genotypic clustering after controlling for individual covariates. However, the trend in higher clustering odds with lower SEP index quartile diminished when additional block-group covariates.

**Conclusions:**

Results stress the need for TB control interventions that take area-based measures into account, with particular focus on poor neighborhoods. Interventions based on area-based characteristics, such as improving case finding strategies, utilizing location-based screening and addressing social inequalities, could reduce recent rates of transmission.

## Background

Although tuberculosis (TB) incidence continues to decline in the United States, studies have revealed that intense TB transmission continues to occur in low-incidence countries [1,2]. To assess transmission dynamics, molecular techniques are used to identify genetic clusters of isolates of *Mycobacterium tuberculosis* with identical genotypes. Those isolates with identical genotypes are thought to indicate recent transmission and a possible continuing transmission chain, while a predominance of unique ‘non-clustered’ isolates implies that most TB cases are caused by reactivation of remote infection [3,4].

Studies have shown that lower socioeconomic status (SES) neighborhoods are correlated with greater clustering among TB strains [5,6] with associations shown between homelessness, unemployment and TB clusters [7-9], yet the association between area-based socioeconomic measures and clustering has not been well assessed. Better knowledge of area-based risk factors for clustering could help develop more effective targeted prevention strategies, and the joint effect of both individual- and community-level measures of SES might help distinguish compositional and contextual effects of socioeconomic factors on TB transmission.

In King County, Washington, the population is highly diverse in terms of birth origin, as well as socioeconomic status. It is likely that TB genotypic clustering would significantly vary, with increased clustering either caused by recent transmission, or by commonly circulating strains within some populations. Those individuals living in block groups with greater socioeconomic disadvantage were hypothesized to be associated with increased TB transmission, as assessed using genotypically-defined TB clusters [8,10].

## Methods

### Study population and setting

The study population consisted of all incident reported culture-TB cases with available genotyping with block group-level geocodes recorded in King County, Washington between January 1, 2004 and December 31, 2008. An incident case of TB was defined according to Centers for Disease Control and Prevention (CDC) surveillance criteria, where TB was either diagnosed for the first time or more than 12 months had elapsed since the patient previously completed TB therapy [11]. A culture-positive sample was defined as isolation of *M. tuberculosis* from a clinical specimen. Patients who did not have both spoligotyping and mycobacterial interspersed repetitive unit-variable-number repeat (MIRU-VNTR) analysis performed on their isolate or did not live in King County at the time of specimen collection were excluded from the analysis. The analysis merged reporting, medical record and genotyping data for TB cases and US census data. Subsequently, only cases with available genotyping results and geocoded addresses were included in the final study population. Approval was granted for this study in May 2009 from the University of Washington and Washington State Institutional Review Boards and final project analysis completed October 2010.

### Data sources

Individual-level case variables were collected at the local level from the Tuberculosis Information Management System (TIMS) and follow standard surveillance definitions [10]. Individual-level variables were subsequently aggregated by block group. Residential address at the time of diagnosis was obtained from patient medical records. Using a geographic information system and latitude/longitude coordinate data, TB cases were geocoded to the corresponding block group of residence. Only block groups with diagnosed TB cases were included in the analyses.

SES was defined at the block group level using census-based indicators of socio-economic disadvantage. A socioeconomic position (SEP) index, was constructed consisting of a standardized *z*-score combining data on percent working class, unemployed, poverty, high school, expensive homes and median household income. To construct the score, each variable was given a standardized score, which was the sum of all block group values with SEP data (n = 1,576), minus the mean sum, divided by the standard deviation, and then summed up the individual *z*-scores. Although high inter-correlations and reliability were noted (Cronbach’s α coefficient 0.78), these measures, along with the index, have previously been used to assess US small area differences in health, with the latter developed based on a factor analysis of eleven single SES factors using rank values of the census data [12]. All socio-economic data as well as area-based data were derived from the US Population Census 2000, SF1 and SF3 [13,14]. All culture-positive patients were genotyped using spoligotyping and 12-locus MIRU-VNTR genotype results obtained through the National TB Genotyping Service. Genotype results were subsequently linked to National TB Surveillance System data using a standardized state case identification number. A cluster was defined as two or more patients with identical TB genotypes within King County. Given the study scope, if cases were part of a Washington cluster designation but unique within King County, they were considered to have a unique TB genotype.

### Statistical analysis

Descriptive statistics were applied to included block groups to assess poverty distributions as well as deviation from King County as a whole. The proportion of TB patients considered to belong to a chain of recent transmission was calculated as the number of subjects belonging to a cluster divided by total number of individuals genotyped [15]. Additionally, the proportion of cases caused by ongoing transmission was estimated using the *n-1* method, where the source case of each cluster was not considered to have recent disease [16]. Incidence rates over time were calculated for both clustered and non-clustered (unique genotype) patients. Univariate associations of independent variables and genotype clustering were assessed using Pearson χ^2^. SaTScan was used to generate a spatial scan statistic identifying geographic areas with a higher-than-expected clustering rate. TB incidence rates were calculated for each SEP stratum by dividing the number of TB cases in a particular quartile by the corresponding stratum population, multiplied by the five years in the reporting period. Cuzick’s nonparametric test for trend across ordered SEP groups was assessed as a summary test of statistical significance [17].

To examine area-level influences on disease clustering in addition to individual attributes, multilevel regression models were used to assess the association between SEP and TB clustering. A two-level hierarchical model with binary clustering outcome was estimated with the high SEP quartile serving as the referent. Hierarchical models have the advantage of yielding accurate parameter estimates and sampling variances in the presence of correlated errors [18]. Prevalence ratios and 95% confidence intervals were estimated by binomial regression with the log link function [19]. **Model 1** consisted of an empty two-level model to examine log-odds of genotypic clustering in an ‘average’ block group and to quantify block-group-level variance. **Model 2** added socioeconomic quartiles as exposure variables. **Model 3** controlled for the individual demographic variables of age, race (modeled as dummy variables with white serving as referent), sex (males as referent) and country of origin (US-born as referent) in addition to SEP index. **Model 4** included individual socioeconomic variables (homelessness with non-homeless referent, employment with employed referent, provider type modeled with dummy variables with public service provision as referent) in addition to demographics and SEP index. **Model 5** added area-level variables of race, ethnicity and foreign birth in addition to individual-level variables and SEP index. Complete case analysis was used such that the number of patients with missing covariates (n = 12) excluded from each model was the same.

## Results

### Block group demographics

The study consisted of 327 block groups in King County with at least one case residing in each (20.7% of block groups with SES data) (Table 1). Block groups included in the study were largely of white (60%), US-born (78%) composition. Hispanic ethnicity made up approximately eight percent of the population, about 10% of individuals were under the federal poverty line and 4% were unemployed. The average five-year incidence rate of TB was 15.6 per 100 000 across all included block groups. In comparison to other block groups in King County (N = 1,249), those included in the study were more likely to contain individuals reporting as black or asian race as well as of Hispanic ethnicity. Additionally, the median proportion foreign-born in these block groups was almost twice as high as that of King County.

**Table 1:** Characteristics of 327 block groups included in the analysis, based on 2000 US Census data

**Block group characteristic**	**Median**	**Mean**	**SD**	**Range**	**King county median**^**b**^
**Demographic variables**					
Population size (persons)	1154	1,302	647	246-4721	1011
Non-Hispanic white race, %	65.5	60.0	23.0	3.1-95.7	79.7
Non-Hispanic asian race,	12.1	15.9	14.3	0-73.2	7.1
Non-Hispanic black race, %	5.6	9.5	10.9	0-56.3	1.8
Hispanic ethnicity, %	5.5	7.7	7.3	0-44.4	3.5
Foreign-born, %^a^	19.3	21.5	13.0	0-62.4	11.7
**Socioeconomic variables**					
Less than HS education, %	9.8	12.8	11.0	0-57.2	6.9
Unemployment, %	3.0	3.6	3.0	0-26.4	2.6
Median household income, $	48 021	51 043	21 297	8667-140 884	56 691
Poverty, %	7.9	10.9	10.0	0-57.5	5.5
Working class, %	57.8	56.4	14.1	21.7-85.4	51.1
Home ownership, %	64.8	59.4	28.6	0-100	73.5
**Tuberculosis measures**					
Tuberculosis mean cases per block group/yr	0.22	0.27	0.20	0.11-1.0	0
Tuberculosis incidence rate per block group (per 100,000 person-years)	12.0	15.6	13.4	3.5-135.5	0

HS, High School; SD, standard deviation.

^a^Excluding US territories and those born abroad to US parents.

^b^King County median reflects all block groups with SES variables available (N = 1576).

### TB patients

Of 686 incident TB cases reported in King County from 2004–2008, 577 (84%) were culture positive, excluding relapses, interjurisdictional transfers, and individuals with missing TB treatment date. Of reported culture-positive cases, 547 (95%) had a reported genotype and 519 (95%) of these cases had both genotyping and block group geocoding available, and therefore were included in the analysis. TB patients were mostly of asian (44%) and black (28%) race, and were largely (81%) foreign-born. Approximately one third of foreign-born patients were identified within five years of arrival in the US.

### Genotype clustering

**Figure 1:**
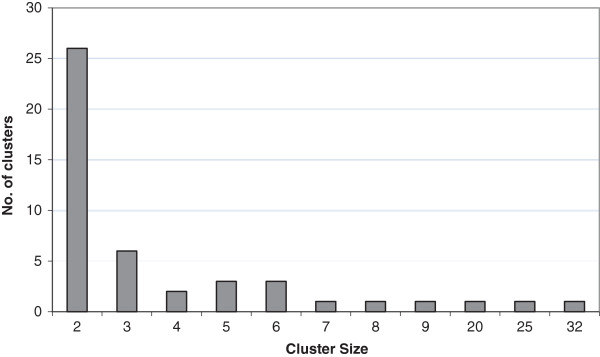
Number of clusters by cluster size.

**Figure 2:**
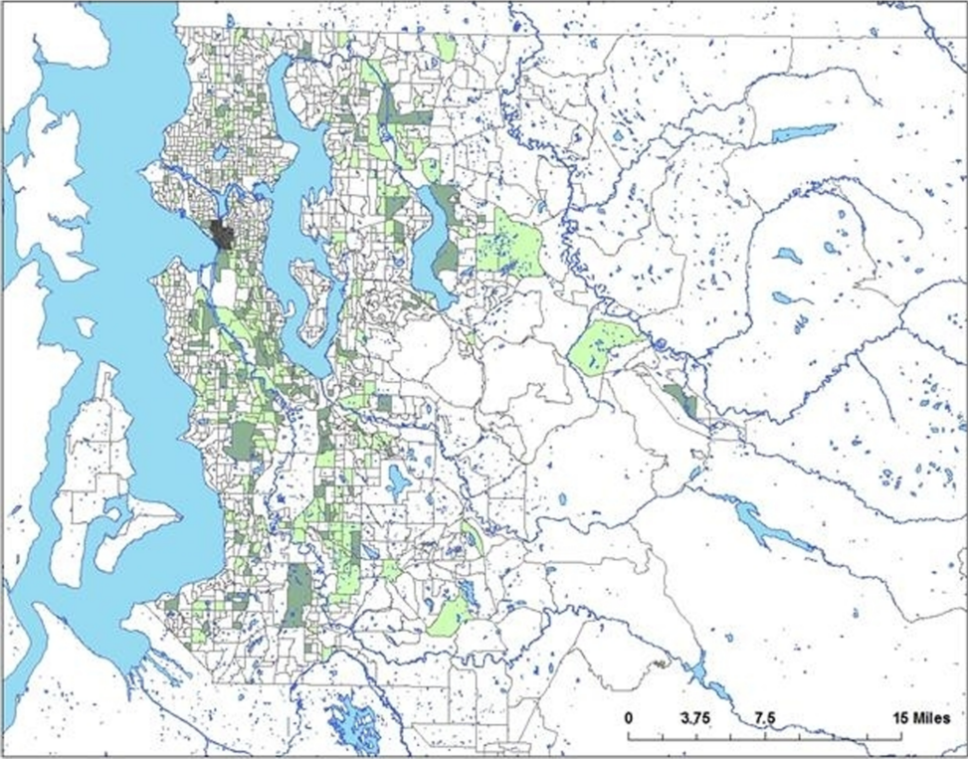
**Genotypic clustering within King County.** Block groups shaded light are those with TB patients; those shaded dark represent block groups with genotypically clustered cases; the significant contiguous block group cluster is shaded in dark grey.

Of those with a known genotyping result, 212 (41%) of isolates clustered genotypically. Forty-six distinct clusters were identified. The number of patients per cluster ranged from 2 to 32 (Figure 1). A median of 3 and mean of 7 patients were identified per cluster. 52 clustered patients (25%) belonged to 2-case clusters and 160 (75%) belonged to clusters with 3 patients or more. Individual clusters ranged in duration from 1 year to the full 5 years of the study period. Based on spoligotype/MIRU match, 336 unique TB genotype strains were identified in King County during this time period. Assuming that 1 patient per cluster resulted from reactivation of remote infection and that the remainder resulted from the spread of recently transmitted disease (n-1 method), 166 (32%) of isolates could be defined as recently transmitted tuberculosis. Further analysis showed that of patients identified after subtracting out the index case and unique genotypes, 134 (83%) matched the isolate of a patient identified within the 1-year period prior to diagnosis date, suggesting potential recent transmission from individual to another. Clustered TB disease was not spatially homogenously distributed throughout the included block groups with significant spatial aggregation of the clustered patients (P = .047 for most likely cluster, Figure 2).

In unadjusted clustering analyses, patients with unique genotyping results were compared to those patients in clusters (Table 2). Clustering was positively associated with female gender, non-Hispanic ethnicity, US birth, homelessness and substance abuse and with indicators of patient infectivity, including pulmonary TB and cavitary TB disease, although not with HIV infection. On average, patients were identified 397 days apart in 2-person clusters, compared with 155 days’ apart among 3-person or greater clusters (P < 0.001).

**Table 2:** **Demographic and clinical features of TB patients included from King County by genotypic clustering of *M. tuberculosis *** **2004-2008**^***a***^

**Patient characteristic**	**Overall**	**Unique**	**Clustered**	**P-value**^**c**^
	**N (%)**^**b**^	**N (%)**	**N (%)**	
**Total**	519 (100.0)	307 (59.1)	212 (40.9)	
**Sex, male**	309 (59.5)	170 (55.4)	139 (65.6)	P = 0.020
**Mean diagnosis age, years**	45.2	46.3	43.5	P = 0.015
				P = 0.055
Age categories				
0-4	3 (0.6)	2 (0.7)	1 (0.5)	
5-14	6 (1.2)	4 (1.3)	2 (0.9)	
15-24	89 (17.2)	51 (16.6)	38 (17.9)	
25-44	180 (34.7)	105 (34.2)	75 (35.4)	
45-64	130 (25.1)	66 (21.5)	64 (30.2)	
65+	111 (21.4)	79 (25.7)	32 (15.1)	
**Race**				P = 0.024
American Indian	14 (2.7)	6 (2.0)	8 (3.8)	
Asian	226 (43.6)	141 (46.8)	85 (40.3)	
Black	145 (27.9)	77 (25.6)	68 (32.2)	
Pacific Islander	18 (3.5)	6 (2.0)	12 (5.7)	
White	109 (21.0)	71 (23.6)	38 (18.0)	
Multiple races	2 (0.4)			
Unknown	5 (1.0			
**Ethnicity**				P = 0.050
Hispanic^d^	56 (10.8)	40 (13.1)	16 (7.6)	
Missing or	3 (0.6)			
Unknown				
**Country of origin**^**e**^				P < 0.001
US-born	101 (19.5)	41 (13.4)	60 (28.3)	
Foreign-born	418 (80.5)	266 (86.6)	152 (71.7)	
**Time from US arrival to TB diagnosis, years**^**f**^				P = 0.123
0-4	156 (37.6)	103 (40.6)	54 (37.8)	
5-9	71 (17.1)	53 (20.9)	19 (13.3)	
10-19	90 (21.7)	55 (21.7)	36 (25.2)	
20+	77 (18.6)	43 (16.9)	34 (23.8)	
Missing	21 (5.1)			
**HIV status, if known**				P = 0.623
Negative	408 (78.6)	231 (93.5)	177 (94.7)	
Positive	26 (5.0)	16 (6.5)	10 (5.4)	
**Previous TB**				P = 0.668
Yes	37 (7.1)	23 (7.7)	14 (6.7)	
No	473 (91.1)	277 (92.3)	196 (93.3)	
Unknown	9 (1.7)			
**Homeless within past year**				P < 0.001
No	452 (87.1)	281 (92.1)	171 (80.7)	
Yes	65 (12.5)	24 (7.9)	41 (19.3)	
Unknown	2 (0.4)			
**Unemployed within past 24 months**				P = 0.613
No	309 (59.5)	180 (58.6)	129 (60.9)	
Yes	210 (40.5)	127 (41.4)	83 (39.2)	
**Substance abuse within past year**^**g**^				P < 0.001
No	442 (87.7)	275 (93.5)	167 (75.5)	
Yes	62 (12.3)	19 (6.5)	43 (20.5)	
**Provider type**				P = 0.059
Health Dept.	411 (79.2)	233 (76.4)	178 (84.8)	
Private provider	35 (6.7)	23 (7.5)	12 (5.7)	
Both	69 (13.3)	49 (16.1)	20 (9.5)	
Missing	4 (0.8)			
**Site of disease**				P = 0.010
Pulmonary	380 (73.2)	212 (69.1)	168 (79.3)	
Extra-pulmonary only	139 (26.8)	95 (30.9)	44 (20.8)	
**Sputum smear result**				P = 0.079
Positive	194 (37.4)	103 (39.2)	91 (47.4)	
Negative	261 (50.3)	160 (60.8)	101 (52.6)	
Not done	63 (12.1)			
Unknown	1 (0.2)			
**Chest radiographic result**				P = 0.386
Normal	90 (17.3)	57 (18.8)	33 (15.8)	
Abnormal	423 (81.5)	247 (81.3)	176 (84.2)	
Not done	5 (1.0)			
Unknown	1 (0.2)			
**Chest radiographic abnormality**^**h**^				P < 0.001
Cavitary	112 (26.5)	52 (21.3)	60 (34.7)	
Noncavitary	305 (72.1)	192 (78.7)	113 (65.4)	
Unknown	6 (1.4)			

^a^Includes only those individuals who are coded within a block group and have an available Spoligotype and/or MIRU genotype result.

^b^Because of rounding, percentages may not total 100.

^c^Compares unique, and clustered groups; Missing, unknown values and multiple values excluded from these comparisons.

^d^Persons of Hispanic ethnicity may be of any race or multiple race.

^e^Foreign-born includes persons born outside the US, American Samoa, the Federated States of Micronesia, Guam, the Republic of the Marshall Islands, Midway Island, the Commonwealth of the Northern Mariana Islands, Puerto Rico, the Republic of Palau, the US Virgin Islands, and US minor and outlying Pacific islands.

^f^Among foreign-born patients.

^g^Substance abuse defined as self-reported excessive alcohol use, non-injection or injection drug use the year preceding TB diagnosis.

^h^Among patients with an abnormal chest x-ray.

**Boldface** indicates significance at P < 0.05.

Among foreign-born patients, average clustered patient incidence rates (5.10/100 000) were lower than average non-clustered (8.93/100 000). The reverse was true among US-born patients, where clustered rates were almost twice as high as non-clustered (7.04/100 000 vs. 4.81/100 000). Greater proportions of foreign-born patients clustered as time between arrival and diagnosis increased (data not shown).

### Socioeconomic trends

**Figure 3:**
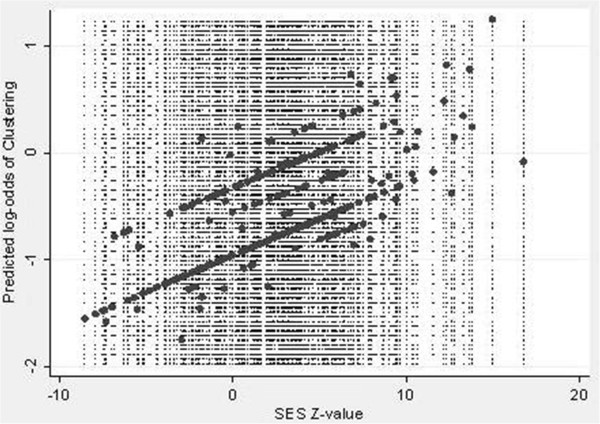
Predicted log-odds of TB clustering by z-score in unadjusted model.

In unadjusted analyses, as SEP decreased, so the proportion clustering increased. A significant linear trend for increased clustering occurred from high to low SEP quartiles (P = 0.001) (Table 3). Clustered case incidence rates increased with lower SEP index, with the greatest increases in rates when going from low to very low SEP quartiles among both clustered and non-clustered cases and with low incidence rates observed among clustered patients living in the highest SEP quartile. Clustered rates were lower than non-clustered for all quartiles, but much more alike in each progressively lower SEP quartile. Unadjusted fitted log odds of clustering for the continuous SES *z*-score are shown in Figure 3. Patients residing in block groups in the lowest 10% of all z-scores were even more likely to cluster (56%).

**Table 3:** Overall incidence rate and clustering by SEP index quartiles

	**High SEP**	**Medium-High SEP**	**Medium-Low SEP**	**Low SEP**
Number of block groups	81	83	82	81
Population, %	26.3	24.8	24.2	24.7
Total case count	104	114	120	181
Total population^a^	1 007 559	950 310	925 794	946 953
5-yr PY Incidence/100,000	10.32	12.00	12.96	19.11
Clustered cases, %^b^	27.9	38.6	44.2	47.5
Non-clustered case 5-yr PY Incidence/100,000^c^	7.44	7.37	7.24	10.03
Cluster case 5-yr PY Incidence/100,000^c^	2.88	4.63	5.72	9.08

SEP = Socioeconomic Position, PY = Person-Year.

^a^Population figure provides proportion of total population in block groups in a particular SEP quartile.

^b^Chi squared test of proportions, top quartile vs. bottom quartile, P < .0001.

^c^Chi squared test for trend across SEP quartiles, P = 0.001.

The majority (73%) of US-born patients clustered at the lowest socioeconomic quartile. Within the low and lowest SEP index quartile block groups, US-born patients were significantly more likely to cluster than foreign-born. Clustering increased significantly with residence in progressively lower SEP block groups among both US- (P-trend 0.005) and foreign-born TB patients (P-trend 0.016).

When stratified by SEP index quartiles, the only significant difference between patients stratified by time from arrival to TB diagnosis was seen among those living in the highest SEP group, where clustering peaked among individuals who had been in the US between 10–19 years from arrival to TB diagnosis (data not shown). Individuals who arrived more recently (0–4 years) were more likely to cluster if they lived in lower SES quartile block groups (P-trend 0.035).

Multilevel models in which community-level characteristics were measured at the block group level demonstrated that lower SEP index was positively associated with TB genotypic clustering after controlling for individual covariates, but the trend of higher clustering risk with lower SEP quartile was diminished when adding additional block-group covariates. In an unadjusted model, a large change in between-community variance (25% decrease) suggested the distribution of SEP quartiles was different across block groups. With progressively lower SEP index quartiles, odds of TB clustering increased compared to the next highest quartile (Table 4, model 2). A positive linear trend was observed (P = 0.005). Once individual demographic variables were included in the model (model 3), the association between SEP and TB clustering did not change. Foreign-born patients were significantly less likely to have clustered disease when compared to US-born patients. Addition of individual-level SES measures did not affect the SEP-clustering association (model 4).

**Table 4:** Odds ratios for associations of TB clustering with individual- and block group-level variables

	**Model 1**	**Model 2**	**Model 3**	**Model 4**	**Model 5**
	**OR (95% CI)**	**OR (95% CI)**	**OR (95% CI)**	**OR (95% CI)**	**OR (95% CI)**
	**N = 507**	**N = 507**	**N = 507**	**N = 507**	**N = 507**
**Block group variance (SE)**	0.54 (0.30)	0.40 (0.28)	0.31 (0.27)	0.31 (0.27)	0.16 (0.25)
Highest SEP		Reference	Reference	Reference	Reference
Medium-High		1.60 (0.94, 1.68)	1.80 (0.99, 1.76)	1.80 (0.99, 1.77)	1.67 (0.93, 1.74)
Medium-Low		2.02 (1.09, 1.82)	1.96 (1.05, 1.81)	1.78 (0.98, 1.76)	1.54 (0.84, 1.72)
Lowest SEP		2.31 (1.21, 1.87)	2.44 (1.22, 1.91)	2.37 (1.19, 1.90)	1.84 (0.92, 1.85)
P-Trend		P = 0.005	P = 0.006	P = 0.012	P = 0.244
**Individual-level demographic**					
Age			0.89 (0.82, 1.04)	0.94 (0.85, 1.09)	0.97 (0.86, 1.11)
American Indian			1.02 (0.42, 1.78)	1.04 (0.42, 1.79)	0.98 (0.40, 1.77)
Asian			1.76 (1.02, 1.71)	1.89 (1.05, 1.76)	2.23 (1.14, 1.87)
Black			1.76 (1.00, 1.73)	1.91 (1.05, 1.78)	1.78 (1.00, 1.75)
Pacific Islander			5.04 (1.32, 17.81)	6.58 (1.44, 24.62)	5.68 (1.34, 20.44)
Female sex			0.71 (0.60, 1.03)	0.75 (0.62, 1.07)	0.72 (0.60, 1.05)
Foreign-born			0.28 (0.24, 0.60)	0.28 (0.23, 0.59)	0.28 (0.23, 0.60)
**Individual-level SES**					
Homeless				1.02 (0.83, 1.21)	0.98 (0.80, 1.20)
Unemployed				0.85 (0.67, 1.16)	0.82 (0.65, 1.15)
Private provider				0.77 (0.69, 1.02)	0.78 (0.70, 1.03)
**Block-Group level demographic**					
Asian					0.78 (0.70, 1.01)
Black					1.25 (1.01, 1.29)
Hispanic					1.09 (0.93, 1.18)
Foreign-Born					1.01 (0.83, 1.20)

OR = Odds Ratio, CI = Confidence Interval, SE = Standard Error, SEP = Socioeconomic Position.

When area-level demographic variables were added, SEP-TB clustering odds ratios decreased in the lowest SEP quartile and the significant linear trend showing increasing with decreasing SEP disappeared (P = 0.244). Areas with larger proportions of black inhabitants were more likely to have TB clusters (Adjusted OR = 1.25; 95% CI: 1.01, 1.29) (model 5). In this multilevel analysis, the only individual-level variables to remain independently associated with TB clustering were foreign-born and race after inclusion of all covariates. These findings suggest that area-level demographic measures, and hence factors related to the area of residence, may substantially affect genotyping clustering among TB patients in the lowest SEP quartile.

## Discussion

In this study, TB genotype clustering was common and closely linked to lower block group socioeconomic status. These findings were novel, in use of a validated SEP index and in showing the explicit association between SES and transmission across areas using a multilevel framework**.** Both clustered and non-clustered case incidence rates were seen to increase with lower SES quartile, with those patients living in the lowest SEP quartile at measurably higher risk for clustering. The analysis confirmed previous molecular epidemiologic investigations identifying patients of US birth, Hispanic ethnicity, homelessness and higher frequencies of substance use as at greater odds for clustering [3,7,20]. As in previous work, there was less evidence of genotypic clustering among foreign-born persons, and genotyping clusters indicated almost no transmission between US and foreign-born groups [20-22]. These findings also confirm similar multilevel analyses that found a positive association between low SES and TB burden and incidence [23,24].

Previous ecologic studies have observed that clustering is greater in poorer areas [5,6,10] and associations have been demonstrated between homelessness or unemployment and TB clusters [20-22]. Clustering by restriction fragment length polymorphism insertion sequence 6110 (RFLP-IS6110) has also previously been shown to correlate with individual markers of low SES, such as homelessness and low income clustering [3,25]. In this study, while individual-level SES measures were crudely associated with clustering, and likely mediate the relationship between SEP and clustering, these measures may have been too crude to pick up the association in the multivariate analyses. Living in a poorer neighborhood may result in higher rates of recent TB transmission because of shared airspace through population density and lack of ventilation [26]. Additionally, contextual effects such as health care availability, or the natural or structural environment may influence transmission [27]. Several studies have also shown that residents of neighborhoods with higher poverty rates encounter environments conducive to stressors and riskier behavior [28-30].

In this study, clustered TB genotypes were spatially aggregated, confirming previous findings that utilized different genotypic and spatial methods [6,31]. In multivariate analyses, neighborhoods which had lower socioeconomic status exhibited greater odds of genotypic clustering. Block-group level race, ethnicity and foreign birth measures attenuated observed associations in the lowest SEP quartile, and may indicate that the effect of neighborhood disadvantage does not dominate that of population demographic characteristics in that area. On the other hand, collinearity between degree of poverty and predominantly minority neighborhoods may make it difficult to disentangle these variables at the block group level. Race has controversially been hypothesized to be the main driving factor in the spatial organization of urban areas, rather than class [32]. However, race may have less of an effect on clustering and ongoing transmission as it does on baseline incidence. SES has been shown to account for much of the increased TB risk attributed to particular races. It is also possible that low SES may not capture all differences in socioeconomic conditions across neighborhoods that also differ in racial/ethnic composition [33].

Previous US-based studies have shown only 25-42% of patients in genotypic clusters to have known epidemiologic links [25,34]. Thus, certain shared genotypes may represent older, endemic strains that are dispersed widely in the US or countries of origin, and clustering may be a result of common contact from circulating strains within a community rather than ongoing active transmission [9]. Spatial variations of unique TB strains by zip code suggest that immigrant neighborhoods have higher rates of unique isolates, suggestive of remote transmission [35]. Some groups of immigrants might share strains acquired in high incidence settings, where one predominant strain type exists. Within each quartile of SEP index, as proportion of foreign-birth in the block group increased, so clustering decreased, perhaps because of higher likelihood of remote TB, or because of decreased stressors as a result of social status, social networks and cohesion [36].

Even if clustering does not indicate an ongoing contagious process, immigrants from areas with known common strains are more likely to be poor and to settle in poorer neighborhoods [37]. Poverty is likely to result in inadequate access to health care and TB treatment [38]. Nevertheless, poverty rates among immigrant groups decline quickly with time in the US [39]. Lower clustering rates among recent foreign-born arrivals in the Unites States reflect a lack of ongoing transmission regardless of SES group. Among foreign-born persons, within the recent arrival group, clustering seemed to increase with lower socioeconomic quartile, but this trend was not observed among those who had been here longer. Genotyping has previously indicated ongoing transmission among the foreign-born within the largest high-incidence zone in Montreal, correlating with lower SES neighborhoods as well as these findings [40]. Previous research has also suggested that new transmission could be expected to cause more active TB in “TB-naïve” neighborhoods, as high prevalence of latent TB infection among foreign-born patients is protective against recurrent TB exposure [41]. Multivariate findings were consistent with this hypothesis. One might also expect less clustering in an area with high migration and strain diversity since isolates not truly linked by new transmission are likely to be distinct [42].

Estimates of degree of clustering and size of clustering are likely to be conservative because individuals with the same genotype are potentially present outside of the study area [43]. Substantial challenges also remain in interpreting the extent of recent transmission, given the background heterogeneity of genotypes, strain evolution over time, and which criteria are used to infer transmission. Authors have previously evaluated various transmission indices in this evolving field of study [44]. Additionally, although the use of spoligotyping and MIRU techniques are currently used by the CDC to determine recent transmission, their low calculated specificities compared to RFLP-IS6110 have been shown to lead to misclassification of patients, inflated estimates of TB transmission, and low positive predictive values [45]. Since 2009, 24-locus MIRU-VNTR has been used in the US and may reduce this misclassification [46]. Finally, some strains may be more transmissible than others, giving rise to sputum smear-positive disease, slower onset of clinical symptoms even as the patient is infectious, or leading to more virulent disease [47].

## Conclusions

Further investigation needs to show how risk factors for clustering are associated with poverty in underlying communities at risk. Substance abuse and homelessness were associated with clustering in this study in unadjusted analyses. Clustering was not associated with HIV infection, as opposed to other recent findings [48] and may demonstrate that in this population co-infected cases were more likely due to reactivation of latent infection rather than re-infection. These findings may also have occurred because HIV-infected TB patients are on average less likely to be the source of transmission, differing demographic profiles, a masking effect due to low force of infection, or the small sample and low prevalence of HIV-infected persons in this study population [49].

Future studies might incorporate other evidence to determine the effect of area-based socioeconomic status on transmission patterns, such as investigating drug susceptibilities and epidemiological linkages that include spatial and temporal associations [48], [50]. Since patient residence at diagnosis seems to be a factor in determining clustering, it would be useful to determine whether clusters are proximal to homeless facilities, bars, or other historically important sites of tuberculosis transmission [51].

The findings reported here suggest the importance of understanding not only individual characteristics of patients leading to clustering but also contextual characteristics of neighborhoods. Results of this study stress the need for TB control interventions that focus on high-risk groups within poor neighborhoods. Recently transmitted disease is most likely propagated among a core of hard-to-reach patients in these areas [5,51]. Poverty is likely to concentrate risk factors for TB and limit access to adequate care, fueling transmission. Interventions based on area-based characteristics, such as improving case finding strategies, utilizing location-based screening and addressing social inequalities, could reduce recent rates of transmission.

## Competing interests

The authors declare that they have no competing interests.

## Authors’ contributions

EO designed the study and performed the data collection. EO, MN and JM analyzed the data. EO, MN, CN discussed core ideas for the paper. All the authors (EO, MN, CN, JM) participated in the writing, reading and approval of the final manuscript.

## Pre-publication history

The pre-publication history for this paper can be accessed here:

http://www.biomedcentral.com/1471-2334/14/227/prepub
